# Optimal position of tendon transfer in reverse shoulder arthroplasty with L'Episcopo for better rotation range of motion

**DOI:** 10.1016/j.jseint.2025.01.007

**Published:** 2025-02-06

**Authors:** Hideki Kamijo, Nobuyasu Ochiai, Eiko Hashimoto, Yohei Shimada, Shohei Ise, Yusuke Matsuura, Seiji Ohtori

**Affiliations:** Department of Orthopedic Surgery, Graduate School of Medicine Chiba University, Chiba, Japan

**Keywords:** Reverse total shoulder arthroplasty, L’Episcopo, Biomechanics, Tendon transfer, Tendon transfer position, Optimal position

## Abstract

**Background:**

Reverse total shoulder arthroplasty (rTSA) with the modified L’Episcopo procedure is useful for rotator cuff tear arthropathy with combined loss of elevation and external rotation. However, depending on the location of the tendon graft transfer, external rotation may improve but cause a limitation of internal rotation. The purpose of this study was to investigate the optimal position of tendon transfer in rTSA with the modified L'Episcopo procedure for better range of motion in rotation using fresh frozen cadavers.

**Methods:**

Eight fresh frozen cadaveric shoulders underwent rTSA with the modified L'Episcopo procedure. To investigate the location of tendon transfer that obtains better internal and external rotation, 6 tendon transfer locations were set and the internal and external rotation at each location was measured with the arm at the side position. The 6 locations were divided horizontally into 3 locations of 225°, 270°, and 315° medial to the bicipital groove. In the lateral view, the height was divided into 2 locations which were the lower end level of the insertion of the teres minor muscle and the middle of the original insertion of the latissimus dorsi muscle.

**Results:**

Three positions were significantly better for external rotation: the height of the lower edge of the teres minor insertion at 270° and 315° from the bicipital groove and the middle of the latissimus dorsi insertion at 315° from the bicipital groove compared to the height of the middle of the latissimus dorsi insertion at 225° from the bicipital groove. On the other hand, for internal rotation, the lower edge of the teres minor insertion and middle of the latissimus dorsi insertion at 315° from the bicipital groove significantly limited internal rotation compared to the other transition positions.

**Conclusion:**

The position of tendon transfer 270° from the bicipital groove at the height of the lower end of the teres minor insertion allowed both relatively good external rotation and internal rotation. Significant limitation of internal rotation was found with transfer 315° from the bicipital groove.

Reverse total shoulder arthroplasty (rTSA) is a useful technique for rotator cuff tear arthropathy, with good postoperative clinical outcomes.^1^^,^^5^^,^^8^^,^^14^ There are many cases in which flexion and internal/external rotation are markedly reduced with cuff tear arthropathy, and although rTSA improves flexion, it is difficult to improve internal and external rotation.^14^^,^^15^ The modified L'Episcopo procedure,^4^ which transfers the tendon of the teres major and latissimus dorsi muscles in combination with rTSA, has been advocated to acquire better active external rotation in cases with combined loss of elevation and external rotation. In previous studies, the position of tendon transfer varied widely, including the attachment of the teres minor,^2^^,^^9^^,^^10^^,^^12^^,^^13^ the height of the attachment of the original level of the latissimus dorsi muscle^3^^,^^4^^,^^6^^,^^17^^,^^18^ on the surgical neck of the humerus,^7^ or suturing the transferred tendon to the stump of the pectoralis major tendon.^3^^,^^6^

It is widely known that conventional rTSA without the use of the L’Episcopo procedure often leads to limitations in internal and external rotation.^14^^,^^15^ It is not clear whether the loss of internal and external rotation is by overtensioning of the transferred tendon after the combined procedure of rTSA with the modified L'Episcopo procedure, or by rTSA itself.^4^ Although Boileau et al speculated that the limited internal rotation could be at least partially explained by excess tension in the transferred tendons and a subsequent tethering effect,^4^ this has not been investigated biomechanically using cadavers. It is important to find the optimal position of the tendon transfer to obtain better internal and external rotation. The purpose of this study was to investigate the optimal position of tendon transfer in rTSA with the modified L'Episcopo procedure using fresh frozen cadavers.

## Materials and methods

### Specimen preparation

This study was approved by our institute’s investigation review board (institutional review board number: 4187). We used 8 fresh-frozen cadaveric upper extremities. They were 4 matched pairs consisting of 4 male specimens provided by the Clinical Anatomy Laboratory of Chiba University. Originally, we thought that using 8 different donors would be preferable; however, due to a limited number of cadavers, we could only secure 4, so we used 4 matched pairs. The age of the cadavers at death ranged from 80 to 95 years, with a mean age of 86 years. They were preserved and stored at −20°C according to the standard operating procedures of the Chiba University School of Medicine and brought to room temperature half a day before autopsy. After thawing, the skin and subcutaneous tissues were removed. The deltoid muscle, pectoralis major, and conjoint tendons were retained, while the supraspinatus, infraspinatus, and teres minor muscles were excised to establish a model of massive rotator cuff tear during the procedure. The shoulder of each corpse exhibited no deformation of the glenoid fossa, history of upper extremity surgery, or history of fractures or other trauma. Each specimen consisted of the shoulder, clavicle, and whole scapula. After the forearm axis was confirmed, the humerus was cut approximately 5 cm proximal to the humeral epicondyle to insert a rod for connecting the torque wrench within the axis of the humerus.

### Procedure of reverse total shoulder arthroplasty

Each shoulder was implanted with the Aequalis Ascend flex reversed shoulder prosthesis (Wright Medical, Memphis, TN, USA) with an onlay humerus design.^19^ Using a delto-pectoral approach, the supraspinatus, infraspinatus, and teres minor muscles were resected, and the long head of the biceps tenodesis was performed by suturing the tendon to the pectoralis major tendon after resecting the intra-articular portion of the biceps. The subscapularis tendon was peeled from the lesser tuberosity of the humeral head, and the capsule and labrum were resected. Osteotomy of the humeral head was performed using a cutting guide with the neck-shaft angle of 145°. Then, a 25-mm diameter baseplate was positioned with its lower edge matching that of the glenoid, ensuring the central post remained parallel to the scapular spine. The baseplate was fixed to the glenoid with 4.5-mm nonlocking screws superiorly and inferiorly and 4.5-mm locking screws anteriorly and posteriorly. To minimize the effect of lateralization on internal and external rotation at the glenoid side, a 36-mm glenosphere was used in all cases. The humeral component size was selected using fluoroscopy.^19^ Humeral retroversion was 20° based on the guide set perpendicular to the epicondylar axis of the humerus. The trial stem was first inserted into the humerus, and it was temporarily removed before performing the tendon transfer.

### Modified L’Episcopo procedure

**Figure 1:**
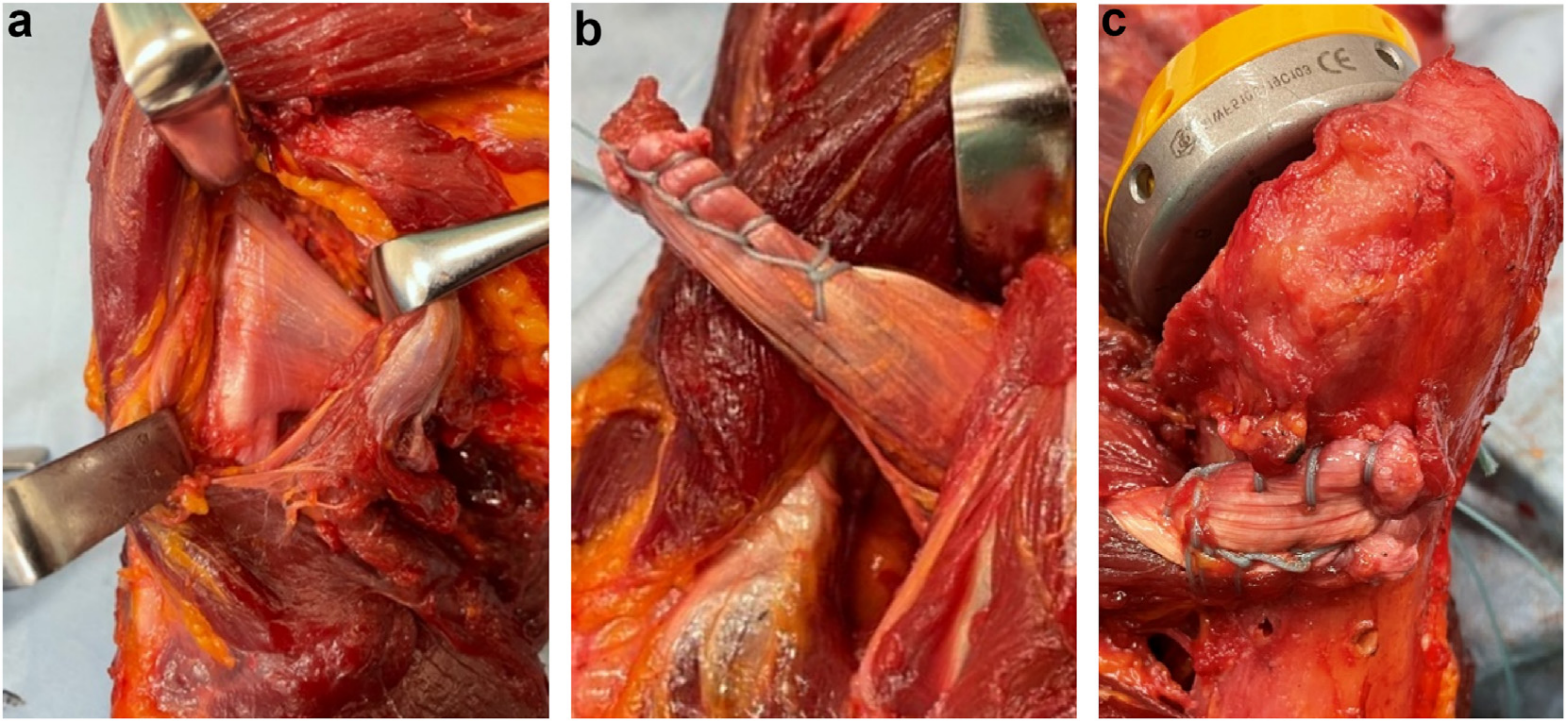
(**a**) Before stripping off the latissimus dorsi and teres major, (**b**) the disconnected transected end of the teres major and latissimus dorsi from the humeral attachment were combined. (**c**) The combined tendon was turned posteriorly on the humerus with a baseball suture. Then, a hole was drilled with K-wire at the site of the tendon transfer, the baseball suture was threaded, and the suture was ligated at the bicipital groove using a metal button.

The teres major and latissimus dorsi muscles were carefully and completely stripped from the humeral attachments with the pectoralis major muscle preserved by pulling the pectoralis major muscle up and down with a retractor. The transected ends of the teres major and latissimus dorsi, detached from their humeral attachment, were whip-stitched together, and the tendons were passed to the posterior aspect of the humerus (Fig. 1).

**Figure 2:**
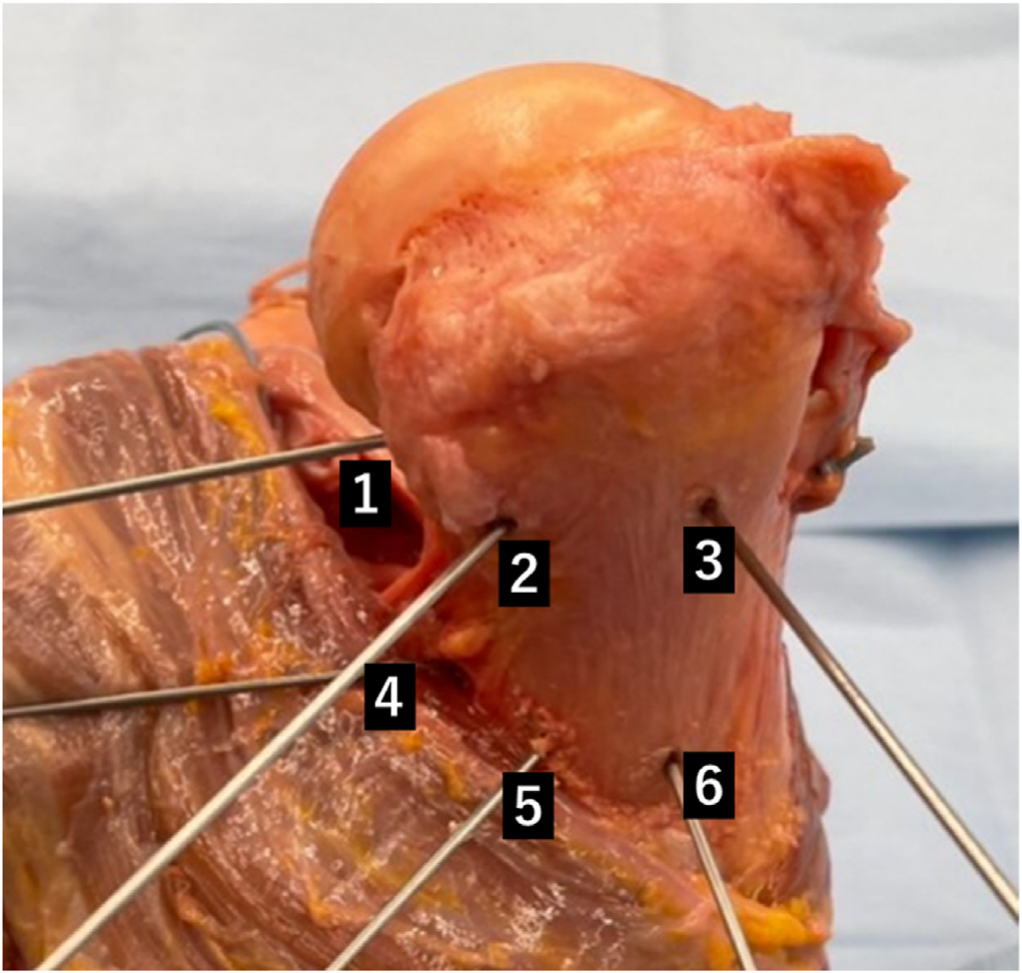
Lateral view of the positions of the tendon transfer.

**Figure 3:**
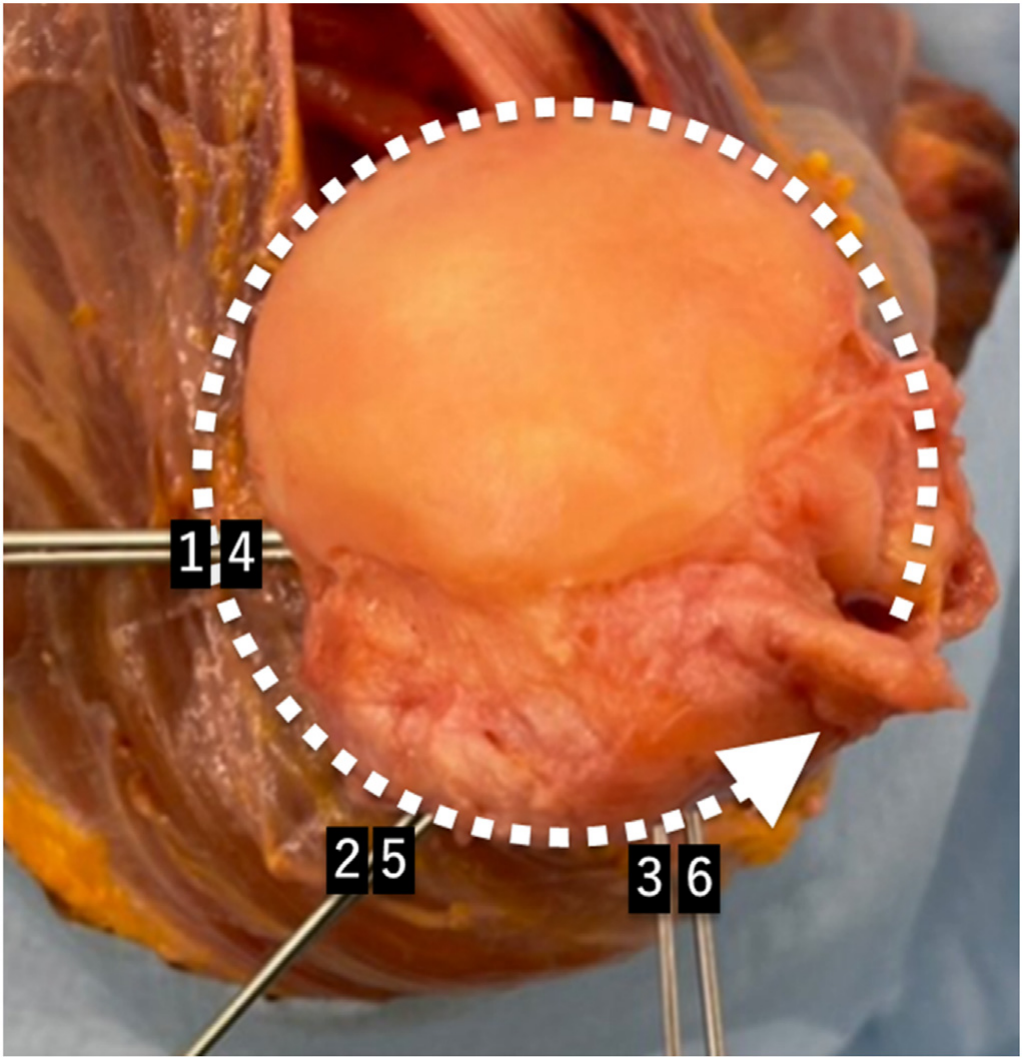
Top view of the positions of the tendon transfer. 1: Lower level of the teres minor insertion 225° from the bicipital groove. 2: Lower level of the teres minor insertion 270° from the bicipital groove. 3: Lower level of the teres minor insertion 315° from the bicipital groove. 4: Middle of the latissimus dorsi insertion 225° from the bicipital groove. 5: Middle of the latissimus dorsi insertion 270° from the bicipital groove. 6: Middle of the latissimus dorsi insertion 315° from the bicipital groove.

To investigate the location of tendon transfer to obtain better internal and external rotation, 6 tendon transfer locations were set. The 6 locations were divided horizontally into 3 locations of 225°, 270°, and 315° medial to the bicipital groove with the bicipital groove as the reference point at 0°. The height was divided into 2 locations: the lower end level of the insertion of the teres minor muscle and the middle of the original insertion of the latissimus dorsi muscle (Figs. 2 and 3). Then a hole was drilled with K-wire at the site of each tendon transfer position, and the suture was inserted into the hole, pulled out at the biceps groove and sutured using a metal button.

Then the humeral component was inserted, and a polyethylene liner was impacted on the humeral component. The subscapularis tendon which was peeled off from the lesser tuberosity was tied to the groove using the stay sutures and metal button after the rTSA was inserted and reduced.

### Experimental protocol

The transected ends of the latissimus dorsi muscle, which were separated from the spine and ribs, were fixed in several places to the board so that latissimus muscle tone could be obtained. A 0.5-kg weight was placed on the deltoid, graft tendon, and subscapularis tendon to pull them in the direction of muscle contraction to tone the muscle.

The internal and external rotation at each tendon transfer position was measured with the arm at the side position. A total of 6 tendon transfers were performed, and the range of rotational motion was measured at each tendon transfer position (Figs. 2 and 3).

**Figure 4:**
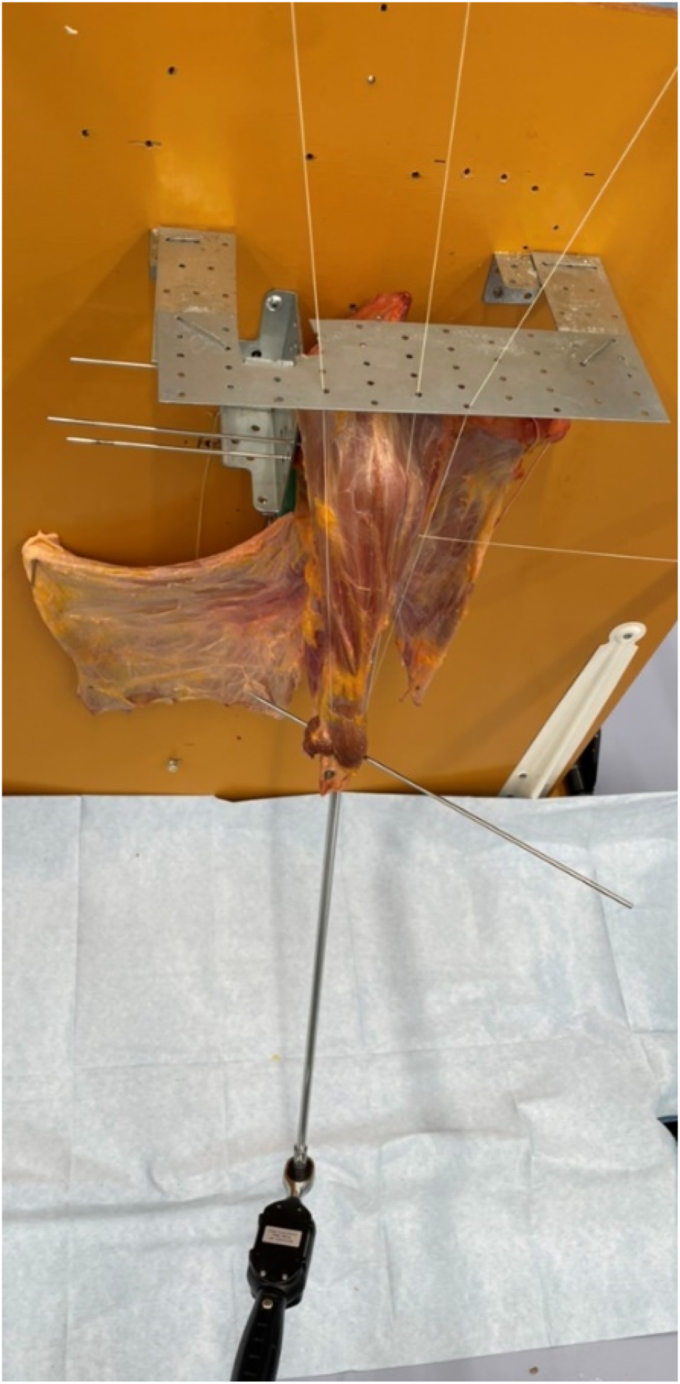
The internal and external rotation angles were measured by manually applying a rotational force of 1 N using a torque wrench.

The angles of internal and external rotation were measured by manually applying a rotational force of 1 N using a torque wrench with the long axis of the scapula protracted 30° anteriorly to the trunk in the horizontal plane as previously described^11^ (Fig. 4). We used a GOYOJO digital torque wrench (GOYOJO Tools, Shenzhen, China) to connect the attachment to an intramedullary rod fixed rigidly within the humeral bone marrow, allowing us to apply internal and external rotation torques in a plane purely perpendicular to the humeral axis with no loss of force. This digital torque wrench can be set to apply forces in the range of 0.3-10 Nm and can be set to emit a high-pitched alarm tone when the set force is exceeded. We tried different turning forces beforehand, and we determined that 1 N is appropriate as the force that is as large as possible within the range of motion that allows the stem moves smoothly without impingement on the scapula or other parts of the body when the humeral shaft is rotated. In this case, the rotation of the bar inserted into the humerus was measured as an angle, which was difficult to measure using electromagnetic or optical tracking. Therefore, we marked a line on the floor that was 120° to the long axis of the scapula and measured the angle between this line and a bar placed through the humerus parallel to the bicipital groove as the actual angle of rotation. The range of motion for internal and external rotation was measured twice each and the average value was recorded. The range of motion of internal and external rotation at each of the 6 tendon transfer positions were compared to each other.

### Statistical analysis

Statistical analysis was performed using a repeated measures analysis of variance followed by a Steel-Dwass post-hoc test using the Bonferroni correction for the multiple comparisons to compare the 6 bony foramen positions at 225°, 270°, and 315° from the groove at the height of the lower end level of the teres minor muscle and at the median level of the vastus lateralis muscle insertion. An alpha level of 0.05 was used for all statistical analyses.

## Results

**Figure 5:**
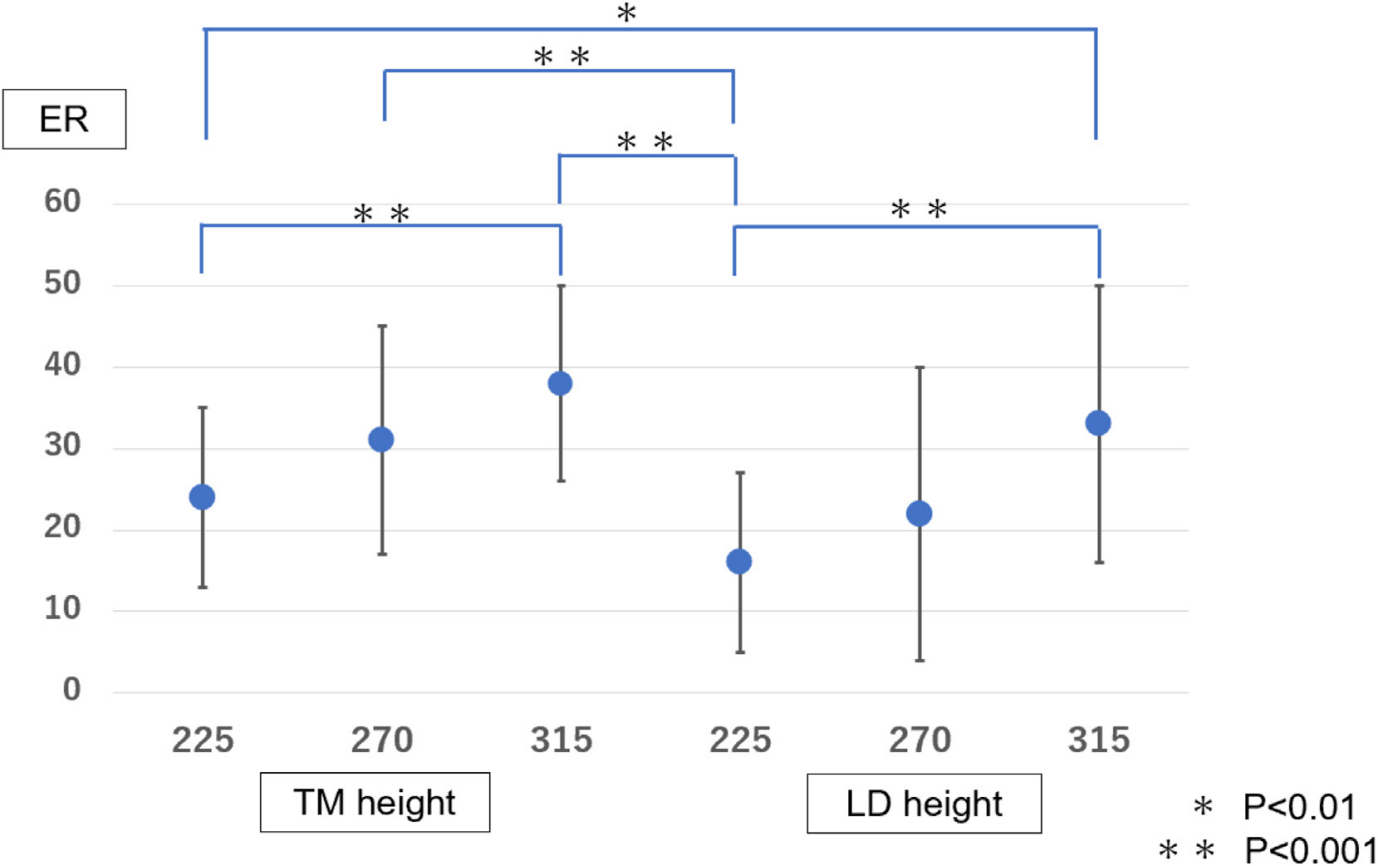
The range of motion of external rotation (Y-axis) was measured and compared at each graft tendon transfer position (X-axis). The height of the lower level of the teres minor insertion at 270° and 315° from the bicipital groove, and the middle of the latissimus dorsi insertion at 315° from the bicipital groove showed greater external rotation compared to the height of the lower level of the teres minor insertion at 225° from the bicipital groove (*P* < .001). *TM*, the lower level of the teres minor insertion; *LD*, the middle of the latissimus dorsi insertion. *TM*, teres minior; *LD*, latissimus dorsi; *ER*, external rotation.

**Figure 6:**
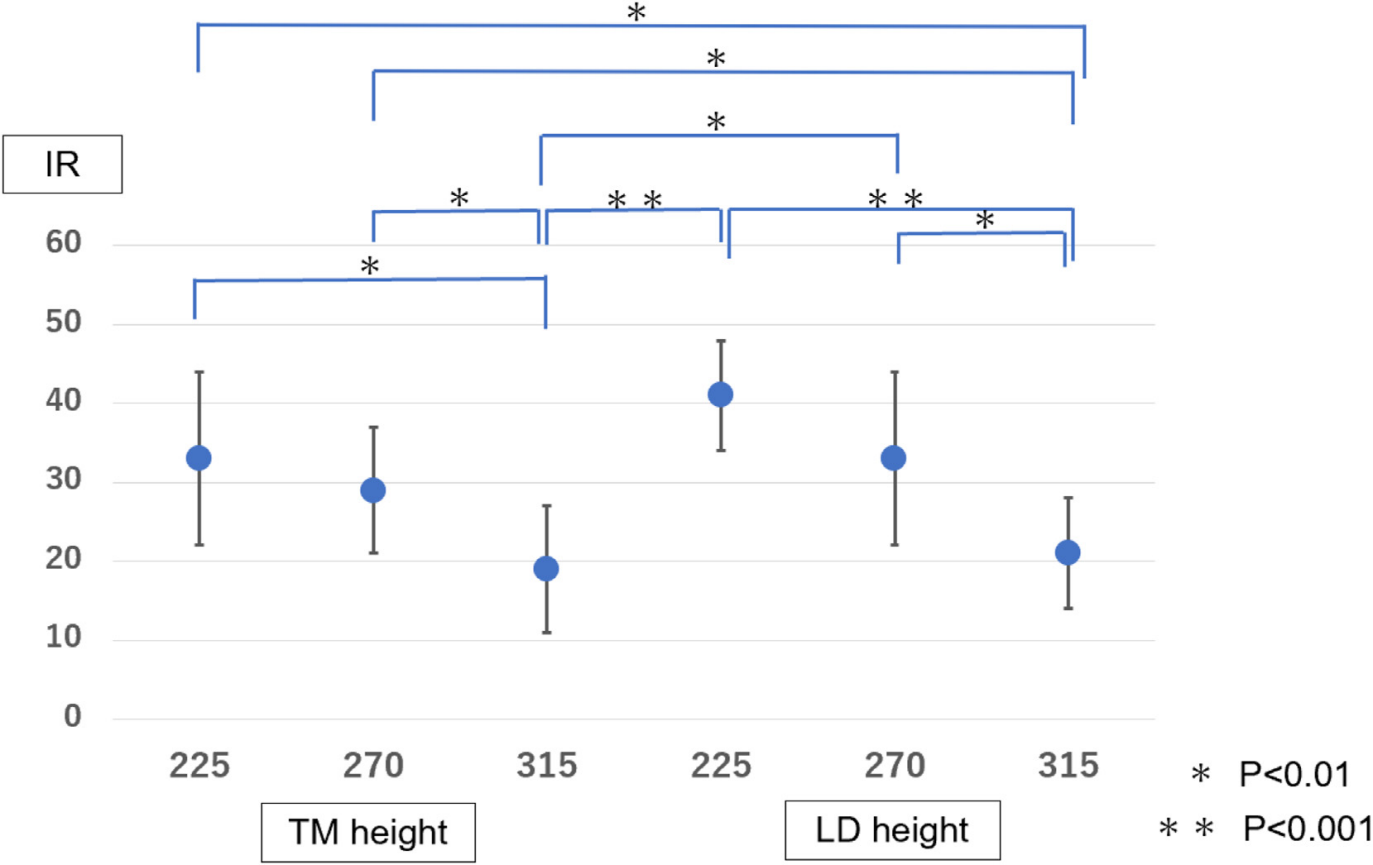
The range of motion of internal rotation (Y-axis) was measured and compared at each graft tendon transfer position (X-axis). More limited internal rotation occurred at 2 positions: 315° from the bicipital groove at the lower level of the teres minor insertion (*P* < .01) and the middle of the latissimus dorsi insertion compared to 225° (*P* < .001) and 270° (*P* < .01). *TM*, teres minior; *LD*, latissimus dorsi; *IR*, internal rotation.

When comparing the 3 tendon transfer positions at the level of the lower teres minor insertion, the 315° position showed significantly better external rotation than the 225° position (*P* < .001) but significantly worse internal rotation (*P* < .01) (Tables I and II, Figs. 5 and 6). Similarly, at the level of the middle of the latissimus dorsi insertion, the 315° position was significantly better than the 225° position in external rotation (*P* < .01) but significantly worse in internal rotation (*P* < .001).

**Table I:** Range of motion of external rotation at each location.

	225°	270°	315°
TM height∗	24 ± 8°	31 ± 14°	38 ± 12°
LD height†	12 ± 13°	22 ± 18°	33 ± 17°

*TM*, teres minor; *LD*, latissimus dorsi.

∗ Lower edge of the teres minor insertion.

† Middle of the latissimus dorsi insertion.

**Table II:** Range of motion of internal rotation at each location.

	225°	270°	315°
TM height∗	33 ± 11°	29 ± 8°	19 ± 8°
LD height†	41 ± 7°	33 ± 11°	21 ± 7°

*TM*, teres minor; *LD*, latissimus dorsi.

∗ Lower edge of the teres minor insertion.

† Middle of the latissimus dorsi insertion.

Overall, the results indicated significantly better external rotation in 3 positions: the height of the lower level of the teres minor insertion at 270° and 315° from the bicipital groove, and the middle of the latissimus dorsi insertion at 315° from the bicipital groove, compared to the height of the middle of the latissimus dorsi insertion at 225° from the bicipital groove (*P* < .001).

**Figure 7:**
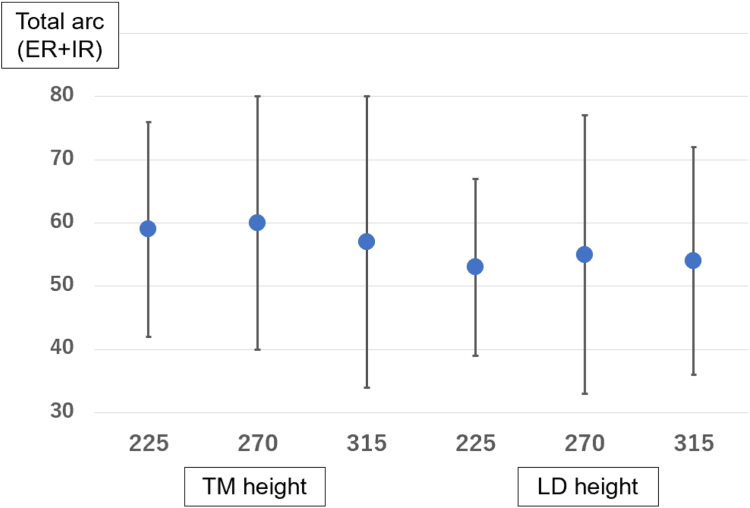
The total arc, the sum of the angles of external and internal rotation (Y-axis) was compared at 6 locations (X-axis). The position of the lower level of the teres minor insertion at 270° from the bicipital groove had the best result with a total arc of 60°, but there was no significant difference in the comparison among the 6 locations. *TM*, teres minior; *LD*, latissimus dorsi; *ER*, external rotation; *IR*, internal rotation.

More limited internal rotation occurred at 2 positions: 315° from the bicipital groove at the lower level of the teres minor insertion (*P* < .01) and the middle of the latissimus dorsi insertion compared to 225° (*P* < .001) and 270° (*P* < .01) (Table II, Fig. 6). Furthermore, the total arc, the sum of the angles of external and internal rotation, was compared at 6 locations. The position of the lower level of the teres minor insertion at 270° from the bicipital groove had the best result with a total arc of 60°, but there was no significant difference in the comparison among the 6 locations (Table III, Fig. 7). Therefore, 270° from the bicipital groove at the height of the lower level of the teres minor insertion was associated with better external rotation without limitation of internal rotation.

**Table III:** Total arc range of motion (external rotation + internal rotation) at each location.

	225°	270°	315°
TM height∗	57 ± 17°	60 ± 20°	57 ± 23°
LD height†	53 ± 14°	55 ± 22°	54 ± 18°

*TM*, teres minor; *LD*, latissimus dorsi.

∗ Lower edge of the teres minor insertion.

† Middle of the latissimus dorsi insertion.

## Discussion

To summarize the results, 270° from the bicipital groove at the height of the inferior end of the teres minor insertion was associated with significantly better external rotation and less limitation of internal rotation, while 315° from the bicipital groove was associated with a significantly larger limitation of internal rotation.

Boileau et al reported in 2007^3^ that directly suturing the transferred tendon that had migrated in a 360° position and pectoralis major muscles caused internal rotation restriction due to excessive tension on the grafted tendon.^3^ Based on these results, in 2010 they modified the position of the grafted tendon insertion in the L’Episcopo procedure to be diametrically opposite the original insertion site of the latissimus dorsi and teres major.^4^ Moreover, they recommended a tendon transfer position close to the teres minor insertion.^2^ In our study, the location of 315° from the bicipital groove is similar to the position of the grafted tendon when sutured directly to the pectoralis major muscle, as originally reported by Boileau et al.^3^ Furthermore, Boileau et al mentioned that it was difficult to identify the cause of the internal rotation limitation after the combined procedure of rTSA with a modified L’Episcopo procedure, whether it is the result of overtensioning of the tendon transfer or because of the rTSA itself.^4^ Our study is the first using fresh frozen cadavers to show that overtensioning of the grafted tendon causes internal rotation limitation.

In our study, external rotation was significantly better without limitation of internal rotation at 270° from the bicipital groove and at the height of the inferior end of the teres minor insertion. Regarding the location, some studies recommend the site of tendon transfer to the teres minor insertion where it might lead to a greater moment arm for external rotation.^3^^,^^10^^,^^12^

Chan et al reported that tendon transfer to the proximal lateral aspect of the greater tuberosity and to the insertion site of the teres minor generated significantly more external rotation torque than transfer to the lateral humeral shaft.^10^ The moment arm analysis by Favre et al also showed that the posterior side of the greater tuberosity (adjacent to the teres minor insertion) produced a greater external rotation moment arm.^12^ Bonnevialle et al reported that, regardless of whether rTSA was performed, the location of the tendon transfer in the L'Episcopo procedure followed the method proposed by Boileau in 2007, placing it at the original height of the latissimus dorsi insertion.^6^ However, subsequently, Boileau et al advocated that it was important to restore external rotation and horizontal balance in the L’Episcopo tendon transfer and recommended in 2017 a tendon transfer position to the teres minor insertion.^2^ The previous studies also showed that the mean humeral distalization produced by rTSA was 33.3 ± 3.8 mm^20^ and Laderman et al reported that mean arm lengthening after rTSA was 1.6 ± 1.9 cm.^16^

Rather than the original insertion height of the latissimus dorsi, it seems more reasonable in terms of maintaining the horizontal level of the latissimus dorsi and teres major to transition toward the teres minor insertion. In this study, the position 270° from the bicipital groove coincided with the attachment site of the teres minor insertion, consistent with previous studies.^2^ This site provided greater external rotation and relatively good internal rotation.

There were several limitations to this study. First, our study was conducted using fresh frozen cadavers, and the results might differ from those of in vivo studies. Second, external and internal rotations were measured only with the arm in the down position; we should have also measured the rotation angles at 30° and 90° abduction positions. However, while previous studies had not examined the limitation of internal rotation following the modified L’Episcopo procedure, our study demonstrated that tendon transfers accompanied by excessive muscle tension could lead to restricted internal rotation. Third, there was potential measurement variability, including errors in the application of torque to the humerus.

## Conclusion

The recommended tendon transfer position for the better range of motion in the modified L’Episcopo procedure is 270° from the bicipital groove to the inferior end of the teres minor insertion. This provides both relatively good external and internal rotation. Significant limitation of internal rotation was found when the site was 315° from the bicipital groove.

## Acknowledgments

The authors thank Wright Medical for their technical support.

## Disclaimers:

Funding: No funding was disclosed by the authors.

Conflicts of interest: The authors, their immediate families, and any research foundation with which they are affiliated have not received any financial payments or other benefits from any commercial entity related to the subject of this article.
